# Molecular mimicry and COVID-19: Potential implications for global fertility

**DOI:** 10.22099/mbrc.2023.47122.1819

**Published:** 2023

**Authors:** Custer C. Deocaris, Malona V. Alinsug

**Affiliations:** Atomic Research Division, Philippine Nuclear Research Institute, Department of Science and Technology, Commonwealth Avenue, Diliman, Quezon City 1101 Philippines

**Keywords:** SARS-CoV-2, Autoimmune diseases, Molecular mimicry, Fertility, MHC-II epitopes, In silico binding

## Abstract

There has been a concerning increase in the incidence of autoimmune diseases following SARS-CoV-2 infection, with molecular mimicry proposed as a potential mechanism. Our study identified nine fertility-associated proteins (AMH, BMP2, CUBN, DNER, ERCC1, KASH5, MSLN, TPO, and ZP3) that exhibit potential molecular mimicry with MHC-II epitopes of SARS-CoV-2 proteins (N, ORF1A, ORF1AB, and S). We screened for epitopes based on in silico binding using DR-, DQ-, and DP-haplotypes that predispose susceptible individuals to autoimmune diseases. Our systematic analysis revealed that 41 countries with population coverage of over 50% had a pre-COVID pandemic total fertility rate of less than 2.1 births per woman. With over 761 million people from 229 countries and territories infected since December 2019, there may be a potential for a foreseeable negative effect on fertility in specific countries, particularly in high-income economies experiencing rapid demographic changes.

## INTRODUCTION

While SARS-Cov-2 infection was initially thought to only cause pulmonary failure, some studies have shown that it can also impact the reproductive systems of both men and women. Due to the presence of viral particles in human sperm and high expression levels of viral entry receptors (ACE2 and TMPRSS2/4) in the ovaries and testis, previous studies have established that the virus can target male and female reproductive organs. Although SARS-Cov-2 originally appeared to be a sexually transmitted infection (STI), a recent meta-analysis demonstrated that this is not the case [1-2]. COVID-19 has been linked to orchitis, low sperm quality, oligozoospermia, testicular inflammation, sperm duct inflammation, and testicular pain in males of reproductive age. Miscarriage, preterm delivery, and extended menstrual cycles have all been associated with SARS-CoV-2 infection in women [3]. A systematic screening of SARS-CoV-2 epitopes revealed that immunoreactive epitopes of the spike (S) glycoprotein share pentapeptides with 27 human proteins related to oogenesis, uterine receptivity, decidualization, and placentation [4]. This suggests that pathogenic cross-reactive antibodies may develop during SARS-Cov-2 infection that could directly impact human fertility. However, the biological relevance of these epitopes remains unexplored.

There have been reports of antigenic mimicry between sexually transmitted infections (STIs) and human proteins. For example, chlamydial proteins share epitopes with human HSP10 and early pregnancy factor (EPF), and some *C. trachomatis *epitopes can generate autoantibodies in the serum and follicular fluid that cause spontaneous abortions in infected female patients with certain DRB1-DQB1 haplotypes [5]. Molecular mimicry by human papillomavirus (HPV) has also been suggested to lead to male and female reproduction-associated problems [4, 6]. While epitopes from STIs and other infectious pathogens have been associated with autoimmune diseases [7], the ‘hygiene hypothesis’ suggests that shared epitopes from infections may also prime the immune system against certain types of cancers and other infectious diseases [8, 9]. In an analysis of immunoreactive epitopes present in SARS-CoV-2 using hexapeptides as antigenic and immunogenic units, we confirm a vast peptide commonality with human proteins involved in human infertility that varies across different populations in terms of MHC II-presentation.

## MATERIALS AND METHODS

**Screening of cross-reactive peptides:** To screen for cross-reactive MHC II-epitopes from the SARS-CoV-2 genome (NCBI:txid2697049), the Immune Epitope Database (IEDB) server according to analytical pipelines from previous studies on molecular mimicry [8, 10] were adopted with some modifications. BLASTP was performed with each SARS-CoV-2 protein against 226,171 human protein sequences in the UniProt database (www.uniprot.org/blast/) using default parameters. Of the 163 peptides identified, 130 sequences (79.7%) have already been reported in the literature [4, 6, 11-14]. The occurrences of each of the 163 sequences from the complete human proteome were then screened using the Peptide Match web interface (www.uniprot.org/peptidesearch/) of UniProt. After filtering out duplicates, a total of 2,440 human proteins were obtained.

**Data mining for human autoimmune proteins:** To identify proteins associated with autoimmunity, the gene names of the 2,440 proteins were queried in Google Scholar, PMC, and PubMed using the keywords “antibody,” “autoimmune,” and “antigen” with Boolean operators. The search process was accelerated by performing batch searches where 200 gene names were concatenated with the Boolean operator “OR.” Using this search strategy, a total of 212 proteins were identified.

**Epitope mimicry screening:** MHC-II associations of each autoimmune-associated protein were determined from the literature. A collection of 65 DR, DP, and DQ haplotypes was used to screen for 15-mer epitopes from SARS-CoV-2 proteins using eight methods for predicting class II epitopes [15]. Because SARS-CoV-2 epitopes in the IEDB database are generally 14-17mer, a peptide length of 15 is a suitable estimate for an average T cell epitope length [16]. This generated a total of 3,910 overlapping epitopes based on a percentile rank of less than 2% for all HLA alleles utilized. After inspecting for occurrences using the identified 163 crossmatching peptides, only 138 (3.5% of 3,910 epitopes) 15-mer peptides containing “unsafe” epitopes (ORF1AB = 45; ORF1AB = 38; N = 14; S = 21; ORF7B = 15; ORF10 = 5), or viral sequences homologous to human proteins within the epitopes were obtained. A total of 56 autoimmune-associated human proteins with unique gene accession containing epitopes from SARS-CoV-2 (ORF1AB=11; ORF1AB=9; N=19; S=14; ORF7B=3) were identified. These proteins were searched in the human autoantigen database of AAgAtlas 1.0 (http://biokb.ncpsb.org/aagatlas/) to confirm their association with autoimmune responses. Finally, to identify if the autoimmune-associated proteins are linked with human fertility, the gene names of the 56 identified proteins were also queried in PubMed using keywords: “patient,” “spontaneous abortion,” “pregnancy,” “placenta,” “testicular,” “miscarriage,” “infertility,” “sterility,” and “ovarian.”

## RESULTS AND DISCUSSION

Out of the 56 autoimmune-associated human proteins screened, nine are either linked or responsible for male infertility, ovarian dysfunctions, infertility, low birth weight, and birth disorders. Sequences of the “unsafe” pentapeptides from SARS-CoV-2 are highlighted in bold and underlined letters (Table 1). Except for three of the seven immune determinants (ORF1AB: DFVEI; S: LIRAA; S: RAAEI), the minimal immune determinants in the epitopes overlap with each other (N: LALLL and ALLLL; ORF1A: LLSVL and LSVLL). A similar indication of cross-reaction due to molecular homology with epitopes was observed from the human papillomavirus (HPV), one of the most common sexually transmitted infectious agents in the general population [17]. The list of epitopes in SARS-Cov-2 reveal overlaps with epitopes from autoimmune-associated human proteins associated with perturbation of reproductive function, both with men and female. The disease association ranges from spontanous abortion, ovarian failure, pre-term, post-natal death and azoospermy (see Supplementary Material for detailed descriptions).

**Table 1:** Epitope mimicry with SARS-CoV-2 proteins and human autoantigens associated with reproductive function and diseases.

**Viral Protein**	**Pos.**	**HLA II Epitope**	**Autoantigen**	**Gene Name**	**Accn No.**	**Reproductive Function**	**Disease-Association**
N	215-234	GDAALALLLLDRLNQLESKM	Delta and Notch-like Epidermal Growth factor-related Receptor	DNER	Q8NFT8	follicle histogenesis and ovarian neovascularization	recurrent spontaneous abortion
N	215-234	GDAALALLLLDRLNQLESKM	Anti-Mullerian hormone	AMH	P03971	regulation of primary follicle transition	premature ovarian failure
N	215-234	GDAALALLLLDRLNQLESKM	Mesothelin	MSLN	Q13421	cell-matrix adhesion (unknown biological function)	premature rupture of membranes (PROM)
N	215-234	GDAALALLLLDRLNQLESKM	Bone morphogenetic protein 2	BMP2	P12643	developmental processes (i.e., cardiogenesis, neurogenesis, and osteogenesis), uterine decidualization	recurrent pregnancy loss
ORF1A	3905-3927	TEAFEKMVSLLSVLLSMQGAVDI	KASH domain-containing protein 5	KASH5	Q8N6L0	meiotic resumption, spindle formation and spermatogenesis	premature ovarian insufficiency; round head and acephalic spermatozoa syndrome
ORF1AB	6743-6767	VCSVIDLLLDDFVEIIIKSQDLSVVS	Cubilin	CUBN	O60494	development of the peri-implantation embryo	reproductive failure in women
S	989-1003	AEVQIDRLITGRLQS	DNA excision repair protein ERCC-1	ERCC1	P07992	Spermatogenesis, oogenesis, functional integrity of germ cell DNA, embryonic organ development, male gonad development	male infertility (azoospermy);
S	1012-1026	LIRAAEIRASANLAA	Zona pellucida sperm-binding protein 3	ZP3	P21754	sperm attachment on the zona pellucida	autoimmune ovarian failure and infertility
S	1007-1028	YVTQQLIRAAEIRASANLAATK	Thyroid peroxidase	TPO	P07202	thyroid hormone biosynthesis for embryonic neuronal development	miscarriage and pre-term birth

Homologous penta- and hexapeptides are shown in bold and underlined letters. Detailed descriptions of fertility-associated autoimmune roles of the proteins are presented in the *Supplementary Material*.

Dotan et al. also identified BMP2 and ERCC1 as potential molecular mimicry targets of SARS-Cov-2. FMN2, an actin-binding protein that mediates intracellular trafficking, invasion, and placentation in human pregnancy, was also enumerated by previous investigators [4]. However, the latter protein was excluded from Table 1 because its shared pentapeptide failed to strongly bind to the class II HLA haplotypes used in the study. The novelty of our approach lies in the increased stringency in selecting molecular mimicry occurrences. This was implemented by carefully validating the shared pentapeptide residing within HLA-restricted epitope regions and cross-checking for human autoimmune-association in the literature.

A clinical validation of autoantibody response to COVID-19 infection was recently published [18]. A comprehensive snapshot of SARS-CoV-2 humoral response revealed elevated IgGs against SPANXN4, STK25, ATF4, PRKD2, and CHMP3 from a cohort of 97 COVID-19 patients in Qatar and New York (USA). Moreover, the authors underscored the effect of COVID-19 infection on human fertility since the most elevated IgG target, SPANXN4, is a vital protein for spermiogenesis. Interestingly, although SPANXN4 was missed from our analysis, ATF4 and CHMP3 were found sharing epitopes with ORF7B and ORF1AB. ATF4 and CHMP3 were excluded since clinical associations of the proteins with autoimmune diseases in the literature were not found. Of note, the infertility-associated proteins cataloged by Dotan et al. were absent in the autoimmune proteome of the patients [4]. These papers collectively provide a mechanism linking cross-reactivity or short peptide sequences derived from infertility-related proteins and proteins in SARS-CoV-2 with observed infertility occurrences and COVID-19 infections.

**Figure 1:**
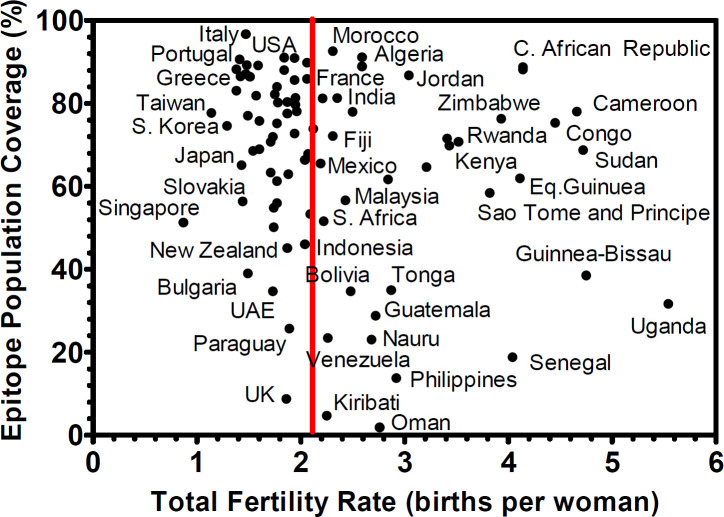
Molecular mimicry by SARS-CoV-2 and human fertility.

The global population coverage of SARS-CoV-2 epitopes was inferred using data from the Immune Epitope Database (IEDB) and the fact that the virus has already infected over 761 million people across 229 countries and territories, as of 06 April 2023. Using IEDB’s population coverage analysis tool, it was found that 50.51% of the global population may respond to the shared epitopes, with 1.01 epitopes recognized by 90% of the world population. Figure 1 shows the projected response to SARS-CoV-2 infertility-associated shared epitopes in 92 countries. There were 41 “endangered” countries with population coverage values greater than 50% and total fertility rates that are below the 2.1 replacement fertility rate, according to the United Nation’s World Population Prospects.

Population coverage by the shared epitopes among 92 countries and territories relative to total fertility rates as of 2020 UN data (https://population.un.org/wpp/Download/Standard/Population/). Class II HLA alleles used: DRB1*01:01, DRB1*01:02, DRB1*01:03, DRB1*04:05, DRB1*04:06, DRB1*03:01, DRB1*03:02, DRB1*03:03, DRB1*03:04, DRB1*07:01, DQA1*05:01, DQA1*05:05, DQB1*02:01, DQB1*03:03, DQB1*06:04, DQB1*06:05. The red vertical line shows the replacement level fertility of 2.1 children per woman, the level of fertility at which a population exactly replaces itself from one generation to the next.

Among the countries with low replacement fertility rates, Italy had the highest population coverage at 99.97%, while the United Kingdom had the lowest at 8.8%. Notably, 31 (75.6%) of these countries are classified as “high-income economies” by the World Bank [19], indicating that this issue may be more prominent in high-income economies with low fertility rates and rapidly aging populations. It should be noted that replacement level fertility needs to be sustained over a sufficiently long period for each generation to exactly replace itself [20].

## Conflict of Interest:

 All the authors declare no conflict of interest. 

## Supplementary materials
