# Supplementary material for: Molecular mimicry and COVID-19: Potential implications for global fertility
Source: Mol Biol Res Commun. 2023;12(2):71–6. doi: 10.22099/mbrc.2023.47122.1819 (PMC10382903; doi:10.22099/mbrc.2023.47122.1819)
Supplement: Supplementary file 1 [file mbrc-12-71-s001.pdf]

Supplementary Material

Descriptions of the identified nine fertility-associated autoimmune human proteins implicated in molecular mimicry with SARS-Cov-2

1. **DNER** is a transmembrane protein that is critical for maternal-embryonic communication leading to appropriate placentation (Lee et al., 2019). During early pregnancy, it binds to Notch1 via cell-cell contact regulating stem cell renewal and differentiation, decidualization, implantation, placentation, and angiogenesis (Haider, Pollheimer, & Knöfler, 2017). Anti-DNER antibodies in serum or cerebrospinal fluid are prevalent in patients with cerebellar ataxia (Campana & Silva, 2021).

2. **AMH**, a protein that regulates steroidogenesis, is a marker for early abortion in the first trimester among women younger than 35 years (Kostrzewa et al., 2020). It controls the growth of the dominant follicle by inhibiting follicle-stimulating hormone (FSH) action (Themmen, 2005).

3. **MSLN,** a 40-kDa membrane-glycoprotein, has been recently identified as a potential blood diagnostic marker for PROM occurring at ≥ 37 weeks of pregnancy. Premature rupture of membranes (PROM) is a common pregnancy complication that frequently leads to maternal and perinatal morbidity (Wang et al., 2018). Commonly associated with several types of cancers, MSLN is also clinically investigated as a target for chimeric antigen receptor T-cell therapy (Faust, Hamill, Kolb, Gopalakrishnapillai, & Barwe, 2022).

4. A transforming growth factor β superfamily member, **BMP2** plays an essential role in organogenesis and placental development. Serum BMP2 levels are elevated during the first trimester of pregnancy but are lower in women experiencing early pregnancy loss (You et al., 2021). The presence of BMP2 autoantibodies in both healthy patients and those with infections linked to type-1 cytokine responses has been noted (Sauerborn et al., 2011).

5. **KASH5** is a component of the LINC (LInker of Nucleoskeleton and Cytoskeleton) complex that connects the nuclear lamina with the cytoskeleton. KASH proteins are essential in male and female gametogenesis (Kmonickova, Frolikova, Steger, & Komrskova, 2020). Functional defects in the protein are associated with a round head and acephalic sperm morphologies where a spermatozoan loses the ability to penetrate the oolemma during fertilization (Syms, Johnson, Lipshultz, & Smith, 1984). The presence of autoantibodies against KASH5 is also associated with early-stage, high-grade serous ovarian cancers (Wilson et al., 2018).

6. **CUBN** is an endocytic receptor that plays a role in lipoprotein, vitamin, and iron metabolism. During embryonic development, CUBN is plays a role in cellular transport of maternally derived nutrients, including high-density lipoproteins, a source of cholesterol, transferrin, retinol, and vitamin B12 (Assémat et al., 2005). CUBN *gene* deletion in mice perturbs the formation of both embryonic and extra-embryonic derivatives, including somites and blood vessels, and leads to embryonic mortality (Smith et al., 2006). Administration of anti-CUBN IgG to pregnant rats induces craniofacial abnormalities, a disorder that resembles holoprosencephaly syndrome in humans (Kozyraki & Gofflot, 2007). Although there is a need for further investigations to support a stronger association with reproductive failure in women, CUBN autoantibody has been detected in the sera of women with recurrent spontaneous abortions, particularly in patient subsets with specific autoimmune conditions (Shoenfeld & Blank, 2004).

7. **ERCC1** is an essential component for nucleotide excision repair and is also involved in recombination repair pathways. The protein is vital for gametogenesis as justified by the general requirement for efficient DNA damage repair in rapidly dividing cells (Hsia et al., 2003). Sera of some SLE patients contain ERCC1 autoantibodies (Sherer, Gorstein, Fritzler, & Shoenfeld, 2004).

8. **ZP3** is among the major glycoproteins of the zona pellucida or egg coat. Some women in infertility clinics have antibodies targeted to zona pellucida. Although there are variations in the incidence of positive results, serum antibodies on zona pellucida or oocyte antigens have been detected in among 7- 68% of infertile women (Luborsky, 2002).The protein has been a target for antifertility vaccines that can block sperm-oocyte interaction (Tung, Ang, & Lou, 1996).

9. **TPO** is essential to the synthesis and secretion of thyroid hormones. TPO autoantibodies are associated with subfertility, recurrent embryo implantation failure, early pregnancy loss, and adverse pregnancy outcomes (Vissenberg et al., 2015).

References

Assémat, E., Châtelet, F., Chandellier, J., Commo, F., Cases, O., Verroust, P., & Kozyraki, R. (2005). Overlapping expression patterns of the multiligand endocytic receptors cubilin and megalin in the CNS, sensory organs and developing epithelia of the rodent embryo. *Gene Expression Patterns, 6*(1), 69-78.

Campana, I. G., & Silva, G. D. (2021). Anti-Tr/DNER Antibody–Associated Cerebellar Ataxia: a Systematic Review. *The Cerebellum*. doi: 10.1007/s12311-021-01346-4

Faust, J. R., Hamill, D., Kolb, E. A., Gopalakrishnapillai, A., & Barwe, S. P. (2022). Mesothelin: An Immunotherapeutic Target beyond Solid Tumors. *Cancers, 14*(6), 1550.

Haider, S., Pollheimer, J., & Knöfler, M. (2017). Notch signalling in placental development and gestational diseases. *Placenta, 56*, 65-72.

Hsia, K.-T., Millar, M. R., King, S., Selfridge, J., Redhead, N. J., Melton, D. W., & Saunders, P. T. K. (2003). DNA repair gene Ercc1 is essential for normal spermatogenesis and oogenesis and for functional integrity of germ cell DNA in the mouse. *Development, 130*(2), 369-378. doi: 10.1242/dev.00221

Kmonickova, V., Frolikova, M., Steger, K., & Komrskova, K. (2020). The role of the LINC complex in sperm development and function. *International Journal of Molecular Sciences, 21*(23), 9058.

Kostrzewa, M., Żyła, M., Garnysz, K., Kaczmarek, B., Szyłło, K., & Grzesiak, M. (2020). Anti-Müllerian hormone as a marker of abortion in the first trimester of spontaneous pregnancy. *International Journal of Gynecology & Obstetrics, 149*(1), 66-70.

Kozyraki, R., & Gofflot, F. (2007). Multiligand endocytosis and congenital defects: roles of cubilin, megalin and amnionless. *Current Pharmaceutical Design, 13*(29), 3038-3046.

Lee, B., Koeppel, A. F., Wang, E. T., Gonzalez, T. L., Sun, T., Kroener, L., . . . Turner, S. D. (2019). Differential gene expression during placentation in pregnancies conceived with different fertility treatments compared with spontaneous pregnancies. *Fertility and Sterility, 111*(3), 535-546.

Luborsky, J. (2002). Ovarian Autoimmune Disease and Ovarian Autoantibodies. *Journal of Women's Health & Gender-Based Medicine, 11*(7), 585-599. doi: 10.1089/152460902760360540

Sauerborn, M., van de Vosse, E., Delawi, D., van Dissel, J. T., Brinks, V., & Schellekens, H. (2011). Natural antibodies against bone morphogenic proteins and interferons in healthy donors and in patients with infections linked to type-1 cytokine responses. *Journal of Interferon & Cytokine Research, 31*(9), 661-669.

Sherer, Y., Gorstein, A., Fritzler, M. J., & Shoenfeld, Y. (2004, October). Autoantibody explosion in systemic lupus erythematosus: more than 100 different antibodies found in SLE patients. In *Seminars in Arthritis and Rheumatism* (Vol. 34, No. 2, pp. 501-537). WB Saunders.

Shoenfeld, Y., & Blank, M. (2004). Autoantibodies associated with reproductive failure. *Lupus, 13*(9), 643-648. doi: 10.1191/0961203304lu2009oa

Smith, B. T., Mussell, J. C., Fleming, P. A., Barth, J. L., Spyropoulos, D. D., Cooley, M. A., . . . Argraves, W. S. (2006). Targeted disruption of cubilin reveals essential developmental roles in the structure and function of endoderm and in somite formation. *BMC Developmental Biology, 6*(1), 1-12.

Syms, A. J., Johnson, A. R., Lipshultz, L. I., & Smith, R. G. (1984). Studies on human spermatozoa with round head syndrome. *Fertility and Sterility, 42*(3), 431-435.

Themmen, A. P. (2005). Anti-Müllerian hormone: its role in follicular growth initiation and survival and as an ovarian reserve marker. *Journal of the National Cancer Institute Monographs, 2005*(34), 18-21.

Tung, K. S. K., Ang, J., & Lou, Y. (1996). ZP3 Peptide Vaccine That Induces Antibody and Reversible Infertility Without Autoimmune Oophoritis. *American Journal of Reproductive Immunology, 35*(3), 181-183. doi: https://doi.org/10.1111/j.1600-0897.1996.tb00028.x

Vissenberg, R., Manders, V. D., Mastenbroek, S., Fliers, E., Afink, G. B., Ris-Stalpers, C., . . . Bisschop, P. H. (2015). Pathophysiological aspects of thyroid hormone disorders/thyroid peroxidase autoantibodies and reproduction. *Human Reproduction Update, 21*(3), 378-387. doi: 10.1093/humupd/dmv004

Wang, Y., Luo, H., Che, G., Li, Y., Gao, J., Yang, Q., . . . Liang, Y. (2018). Placental protein 14 as a potential biomarker for diagnosis of preterm premature rupture of membranes. *Molecular Medicine Reports, 18*(1), 113-122.

Wilson, A. L., Moffitt, L. R., Duffield, N., Rainczuk, A., Jobling, T. W., Plebanski, M., & Stephens, A. N. (2018). Autoantibodies against hsf1 and ccdc155 as biomarkers of early-stage, high-grade serous ovarian cancer. *Cancer Epidemiology and Prevention Biomarkers, 27*(2), 183-192.

You, J., Wang, W., Chang, H.-M., Yi, Y., Zhao, H., Zhu, H., . . . Zhu, Y.-M. (2021). The BMP2 Signaling Axis Promotes Invasive Differentiation of Human Trophoblasts. *Frontiers in Cell and Developmental Biology, 9*. doi: 10.3389/fcell.2021.607332
